# The Trp triad within the V-domain of the receptor for advanced glycation end products modulates folding, stability and ligand binding

**DOI:** 10.1042/BSR20193360

**Published:** 2020-01-31

**Authors:** Venkata S.K. Indurthi, Jaime L. Jensen, Estelle Leclerc, Sangita Sinha, Christopher L. Colbert, Stefan W. Vetter

**Affiliations:** 1 North Dakota State University, Department of Pharmaceutical Sciences, NDSU Dept. 2665, Fargo, ND 58108-6050, U.S.A.; 2 North Dakota State University, Department of Chemistry and Biochemistry, NDSU Dept. 2735, Fargo, ND 58108-6050, U.S.A.

**Keywords:** advanced glycation end products, fluorescence spectrometry, RAGE receptor, S100B, tryptophan residues, X-ray crystallography

## Abstract

The receptor for advanced glycation end products (RAGE) recognizes damage-associated molecular patterns (DAMPs) and plays a critical role for the innate immune response and sterile tissue inflammation. RAGE overexpression is associated with diabetic complications, neurodegenerative diseases and certain cancers. Yet, the molecular mechanism of ligand recognition by RAGE is insufficiently understood to rationalize the binding of diverse ligands. The N-terminal V-type Ig-domain of RAGE contains a triad of tryptophan residue; Trp^51^, Trp^61^ and Trp^72^. The role of these three Trp residues for domain folding, stability and binding of the RAGE ligand S100B was investigated through site-directed mutagenesis, UV/VIS, CD and fluorescence spectrometry, protein–protein interaction studies, and X-ray crystallography. The data show that the Trp triad stabilizes the folded V-domain by maintaining a short helix in the structure. Mutation of any Trp residue increases the structural plasticity of the domain. Residues Trp^61^ and Trp^72^ are involved in the binding of S100B, yet they are not strictly required for S100B binding. The crystal structure of the RAGE-derived peptide W72 in complex with S100B showed that Trp^72^ is deeply buried in a hydrophobic depression on the S100B surface. The studies suggest that multiple binding modes between RAGE and S100B exist and point toward a not previously recognized role of the Trp residues for RAGE-ligand binding. The Trp triad of the V-domain appears to be a suitable target for novel RAGE inhibitors, either in the form of monoclonal antibodies targeting this epitope, or small organic molecules.

## Introduction

The receptor for advanced glycation end products (RAGE, uniprot Q15109) is a cell surface receptor that senses a group of molecules that are commonly summarized as damage-associated molecular patterns (DAMPs). DAMP ligands of RAGE are structurally diverse and include advanced glycation end products (AGEs), for which the receptor is named, members of the S100 protein family [1], high mobility group box 1 protein (HMGB1) [2], nucleic acids [3], phospholipids [4], negatively charged polysaccharides [5], as well as amyloid peptides [6]. Cell biological and animal studies have identified RAGE activation as an important factor in diabetic vascular complications [7], neurodegeneration [8,9], chronic inflammatory diseases [10] and certain cancers [11–13]. Preclinical studies also have provided strong support for the hypothesis that RAGE inhibition could be beneficial to the treatment of these diseases [14]. Despite the relevance of RAGE in multiple diseases, fundamental mechanistic questions regarding DAMP recognition, receptor activation and ligand specificity of RAGE signaling remain unanswered.

The mature human RAGE receptor is a 382-residue cell surface protein with three Ig-like extracellular domains, termed as the V-, C1- and C2-domains; a single transmembrane domain; and a short cytoplasmic tail. The intracellular, C-terminal 44 residue tail is required for RAGE signaling, but the exact mechanism of intracellular signaling activation has not been unambiguously established. Activation of RAGE is initiated by binding of DAMPs to the extracellular domains. Most ligands bind to the N-terminal V-domain [15], while a few ligands also interact with the C1- or C2-domains [16,17]. RAGE ligand-binding studies have often relied on biophysical protein–protein interaction studies such as surface plasmon resonance (SPR) measurements [18], which reveal binding affinities and binding kinetics, but do not provide insights into the structural basis for ligand binding by RAGE. X-ray crystallographic or NMR studies of RAGE and RAGE–ligand complexes have been published for two S100 ligands [16,17,19], DNA [20], heparan sulfate [21] and two AGE-modified peptides [22,23]. While these studies provide insight in the protein–protein binding interfaces, they have not resulted in a standard model for RAGE–ligand recognition that can be applied to all ligands. In fact, some studies have reported contradictory findings. For example, an NMR study has mapped the binding of S100A6 to the RAGE V-domain [16], while a crystallographic study assigned the dominant binding surface for S100A6 to the C1-domain [17]. Thus, alternate techniques and approaches to characterize ligand binding to RAGE will provide complimentary and novel insights in RAGE–ligand interactions.

S100B (uniport P04271) is a prototypical member of the S100 protein family. The S100B dimer contains two pseudo-EF-hand motifs (S100 EF-hands), which bind Ca^2+^ weakly (>350 μM), and two typical-EF-hand motifs, which bind calcium with higher affinity (<50 μM) [24]. Ca^2+^-binding to the high affinity calcium-binding sites induces a conformational change (S100 Ca^2+^-switch) between helix 3 and 4 [25]. As a consequence, hydrophobic amino acid side chains become exposed to the protein surface where they form hydrophobic patches that facilitate binding of S100B target proteins. The role of hydrophobic anchor residues, including Trp side chains, for binding to S100B is well documented. Peptide phage display studies have identified a general motif for S100B binding epitopes centered around a central Trp residue [26]. Sequence comparison of the consensus S100B-binding motif and the amino acid sequences surrounding residues Trp^51^, Trp^61^ and Trp^72^ of the RAGE V-domain indicates that each Trp residue could be a hydrophobic anchor residues for S100B binding.

In addition to hydrophobic interactions, electrostatic interactions play a role in RAGE–target interactions, including S100B binding. This is supported by the observation that highly negatively charged molecules, such as DNA [3] and heparin [27], which do not contain hydrophobic anchor residues or structures, are recognized by RAGE. A model for S100B binding to RAGE based on electrostatic complementarity has been proposed by Fritz [28]. This model suggests that the S100B homodimer binds to a positively charged surface region of a single RAGE V-domain, forming a (S100B)_2_:(RAGE) heterotrimer. A shortcoming of the proposed binding model is that identified interacting residues do not form a continuous surface and that the proposed binding mode does not readily allow for the stabilization of RAGE dimers. Subsequently, a tetrameric form of S100B has been proposed by the same group to bind to and to activate RAGE through formation of a (S100B)_4_:(RAGE)_2_ heterohexamer [29]. A similar contradictory picture has emerged for S100A5 binding to RAGE, where Kim et al. [30] report (S100A5)_2_:(RAGE) heterotrimer formation, while Cho et al. [31] postulate formation of a (S100A5)_2_:(RAGE)_2_ heterotetramer. Thus, NMR and X-ray structural studies suggest that multiple binding modes between RAGE and S100 proteins are possible.

In the present study, the role of the Trp triad (Trp^51^, Trp^61^ and Trp^72^) in the RAGE V-domain for folding, stability and S100B binding was investigated. A combination of site-directed mutagenesis, fluorescence spectroscopy, ligand-binding assays and X-ray crystallography experiments revealed the relative positioning of each Trp residue within the free V-domain, as well as in the V-domain:S100B complex. The results identified significant structural plasticity surrounding residues Trp^61^ and Trp^72^ and the ability of these Trp residues to adopt multiple alignments within the V-domain and its S100B complex. Direct V-domain-S100B-binding studies and the crystallographic analysis of a novel S100B:V-domain peptide complex support the spectroscopic data. The emerging picture for S100B binding to RAGE indicates that a simple lock-and-key binding mode is unlikely for this receptor–ligand pair and that structural plasticity is a key feature for RAGE to function as a multi-ligand receptor.

## Experimental

### Materials

All chemicals were of biochemical grade. Molecular biology reagents were purchased from New England Biolabs. Bacterial growth media components were from EMD or Gibco. Chromatography media were from GE Life Sciences. Chemicals for peptide synthesis were from Peptide International.

### Protein mutagenesis, expression and purification

The V-domain (residues 23–132) of RAGE (uniprot Q15109) was cloned into the pET15b expression vector using the NcoI and XhoI restriction sites. Site-specific mutation of tryptophan to alanine was achieved using the QuikChange site-directed mutagenesis kit (Agilent, Santa Clara). The regions encoding the RAGE domain genes were sequenced to confirm correct mutagenesis and sequence integrity.

Proteins were expressed in the disulfide isomerase expressing *Escherichia coli* strain Shuffle T7 Express (New England Biolabs, Ipswich) in LB medium. Cells were grown at 37°C to an OD_600_ of 0.5–1.0, induced with 1 mM IPTG and the temperature reduced to 30°C. After 4 h, cells were harvested by centrifugation, resuspended in buffer A (50 mM Tris, pH 8.0; 300 mM NaCl; 20 mM imidazole) and stored frozen at −20°C.

Purification of the RAGE domains employed a two-step protocol. The cells were disrupted by sonication on ice with a Misonic XL-2000 sonicator, equipped with a P4 tip. The sonicate was clarified by centrifugation at 17000×***g*** for 20 min and the supernatant was filtered through a 0.45 μm filter prior to loading on to a HisTrap HP column (GE Life Sciences) equilibrated with buffer A and connected to a Bio-Rad Biologic DuoFlow chromatography system. After extensive washing with buffer A, the captured protein was eluted with buffer B (50 mM Tris, pH 8.0; 300 mM NaCl; 200 mM imidazole). The pooled protein fractions were diluted 1:1 with buffer C (50 mM Na acetate, pH 5.5) and loaded on to a cation exchange column (HiTrap SP FF column, GE Life Sciences). The V-domain proteins were eluted with a linear gradient of buffer D containing (50 mM Na acetate, pH 5.5, 1 M NaCl). This step separated the protein fraction from co-purified oligonucleic acids. The purity of the protein was verified by UV/Vis spectrometry and SDS/PAGE. All RAGE domains showed the expected UV/absorbance spectrum and a single protein band at the expected molecular weight (Supplementary Materials and Figure S1).

Recombinant human S100B (uniprot: P04271) was expressed in the plasmid pGEMEX and was purified as described previously [32,33].

### Secondary structure analysis by circular dichroism spectroscopy

Circular dichroism (CD) spectra were recorded on a Jasco J815 spectropolarimeter equipped with a PFD-425S Peltier cell holder in a 1 mm path length cuvette. The concentration for all protein samples was 25 µM in 50 mM Tris, 150 mM NaCl, pH 7.0. The samples were scanned in continuous mode from 180 to 260 nm in 0.5 nm increments, with a scan rate of 10 nm per minute and a digital integration time of 8 s. Ten spectral scans were averaged by the instrument software. For each protein sample, five independent replicate measurements were performed. CD-spectra were deconvoluted using spectral data ranging from 180 to 260 nm using the CONTIN algorithm in the DichroWeb software [34,35]. Statistical analysis of secondary structure compositions was performed by pairwise Student’s *t* test.

### Steady-state fluorescence measurements

Steady-state excitation and emission spectra were recorded on a FluoroMax spectrofluorometer (Horiba Instruments Inc., U.S.A.) using quartz cuvettes with either a 5 mm or a 10 mm path length. Tryptophan was excited at 295 nm to minimize tyrosine excitation. Three scans were recorded and averaged.

### Fluorescence lifetime measurements

Tryptophan fluorescence lifetime measurements of the RAGE V-domain and its mutants were performed using the photon counting fluorohub (Horiba Instruments Inc., U.S.A.) connected to the FluoroMax. The samples were excited at 280 nm with a nano LED. The emission was recorded at 350 and 375 nm to reduce the tyrosine influence. Peak saturation was set to 1000 counts and a 4 nm slit width was used. For fluorescence decay experiments, 1 μM of the protein was used in a quartz cuvette with reduced path length of 5 mm. The effect of S100B binding on fluorescence lifetime of the V-domain Trp mutants was also studied in the presence of 2 mM calcium. To test the effect of guanidinium chloride (GuHCl) unfolding on the decay times, each protein sample was unfolded with 5 M GuHCl overnight and the lifetimes measured. The lifetimes of the samples were analyzed by DAS6 software (Horiba Instruments Inc., U.S.A.) using a two-exponential model. The time-dependent emission intensity traces were analyzed by double-exponential fitting, which yielded a short lifetime component τ_1_ of 1–2 ns and a long life-time component τ_2_ of 4–7 ns. To simplify the interpretation of the fluorescence lifetime data for each sample, the amplitude weighted average lifetime τ_m_ was used [36,37].

### Fluorescence quenching

Acrylamide quenching of tryptophan fluorescence measurements was performed using a protein concentration of 1 μM in quartz cuvette with a 5 mm path length. Acrylamide concentrations were incrementally increased to 0.25 M and a correction factor of *ε*_295_ = 0.25 M^−1^.cm^−1^ was applied to account for the inner filter effect of acrylamide [38]. The samples were excited at 295 nm and the emission spectra were recorded. Stern–Volmer quenching constants, *K*_sv_, were calculated according to F_0_/F = 1+*K*_sv_ [Q], where F_0_ is the fluorescence intensity (FI) in the absence of the quencher Q and F is the FI in the presence of the quencher at the concentration [Q].

### Differential scanning fluorimetry (Thermofluor assay) [39]

SYPRO Orange (Sigma) was obtained as a 5000× solution and initially diluted to 100× in DMSO, then further diluted to 10× in PBS, pH 7.4. This solution was mixed 1:1 with 5 μM V-domain in 2 M ammonium sulfate. The ammonium sulfate was identified as a suitable additive for the thermofluor assays of the V-domains by screening with the Hampton Crystal Screen HR2-100 (Hampton Research). Thermal denaturation was done in 50 μl volumes on a Stratagene Mx3000P qPCR instrument with a customized excitation/emission (492 nm/610 nm) filter set-up. The temperature was increased in 1°C intervals at a rate of 2°C/min. Two independent experiments were performed with five samples of each V-domain variant. The obtained FI versus temperature (T) data were analyzed by calculating the relative change in fluorescence per °C as a function of temperature (ΔFI/ΔT vs. T).

### V-domain binding to S100B measured by fluorescence polarization

Binding of the V-domain mutants to S100B was measured by monitoring the change in FITC fluorescence polarization during titration with each RAGE V-domain. S100B was fluorescein labeled using an FITC labeling kit (Pierce/Thermo Fisher prod# 53027) according to the manufacturer’s guidelines with the modification that a 5-fold instead of a 20-fold excess of FITC relative to protein was used. This was done to reduce the number of fluorescein molecule attached to each S100B protein. A total of 600 μl of 1 µM S100B-FITC solution in 50 mM Tris, pH 7.0 and 2 mM CaCl_2_ was titrated by incremental addition of V-domain stock solutions in a quartz cuvette with reduced path length of 5 mm. After each addition of V-domain, an equilibration time of 2 min was allowed so the increase in sample volume at the end point of the titration was less than 3%. Titrations were repeated three to five times and the best fit for the data was obtained using a 1:1 RAGE:S100B stoichiometry model as described previously [40,41]. Nonlinear least square fitting was done with Kaleidagraph (Synergy Software). The excitation wavelength was 494 nm and the emission wavelength was 518 nm.

### Crystallization of S100B with the W72 peptide

S100B in 25 mM Tris/HCl, pH 7.8; 150 mM NaCl; and 4 mM CaCl_2_ was concentrated by centrifugation to 50 mg/ml. Lyophilized peptide W72 (ac-SPQGGGPWDSVARVL-amide) was resuspended in sterile water to 8 mM. For co-crystallization of S100B and W72, 2 mM S100B was combined with 3 mM peptide and stored on ice for ∼30 min prior to crystallization trials. Crystallization trials were performed using the sitting drop vapor diffusion method. Drops containing a 1:1 ratio of protein-peptide solution and reservoir solution were equilibrated against 500 μl of a reservoir solution containing 0.1 M Na cacodylate, pH 6.8; 22% w/v PEG 3350; and 5 mM CaCl_2_. Crystals were observed within 1 week of incubation at 20°C, and were mounted with MicroMount cryoloops (MiTeGen, Ithaca) prior to immersion in cryo-protectant solution and flash-freezing in liquid nitrogen. The cryo-protectant solution contained reservoir solution plus 20% v/v glycerol.

### Crystal structure determination of S100B:W72 RAGE peptide complex

High-resolution diffraction data were collected at cryogenic conditions (∼100K) with an X-ray wavelength of 0.9792 Å at the Northeastern Collaborative Access Team (NE-CAT) 24ID-C beamline of the Advanced Photon Source (Argonne National Laboratory, Argonne, IL). Crystals belonged to the monoclinic space group *P2_1_* with unit cell parameters, *a* = 35.1 Å, *b* = 59.8 Å and *c* = 47.6 Å, and β = 111.6°. A dataset from a single crystal was processed using NE-CAT’s RAPD automated processing system (https://rapd.nec.aps.anl.gov/rapd), which incorporates XDS [42,43] for integration and scaling. The structure of S100B was determined by molecular replacement with a single monomer of Ca^2+^-loaded human S100B (PDB entry 2H61) in Phaser-MR of PHENIX [44–46]. The W72 peptide model was manually built in COOT [47] by placing a polyalanine chain and mutating residues with strong *2F_o_-F_c_* electron density. The remaining residues were placed based on the W72 peptide sequence. Water oxygen atoms were positioned using PHENIX, with subsequent visual verification. Refinement was carried out in PHENIX. R*_work_* converged to 15.7% and R*_free_* to 18.3%. The final model included two S100B molecules, ten residues of one copy of the W72 peptide, two coordinated Ca^2+^ ions per S100B monomer and 214 water molecules per asymmetric unit. No Ramachandran outliers were present, as determined by MOLPROBITY [48]. All figures were prepared using PyMOL v.1.5.0.4 (Schrödinger). Analysis of surface areas, protein interfaces, assemblies and interactions were determined using the PISA server (http://www.ebi.ac.uk/pdbe/pisa/) [49]. Atomic models and structure factors have been deposited into the Protein Data Bank (PDB entry 5D7F).

## Results

### Trp residues are not required for recombinant expression and folding of the RAGE V-domain

Seven Trp-Ala mutants of the RAGE V-domain (W51A; W61A; W72A; W51A/W61A; W51A/W72A; W61A/W72A; and W51A/W61A/W72A) were recombinantly expressed in *E. coli* and purified. CD spectrometry was used to obtain a detailed characterization of the secondary structure composition of the V-domain and its mutants. Analysis of CD-spectra of the wild-type V-domain showed the secondary structure composition (Table 1 and Supplementary Figure S2) was similar to the previously reported CD secondary structure analysis of the V-domain [50] and a computational secondary structure analysis of the NMR structure of the V-domain (pdb: E2E5) using the program Stride [51].

**Table 1:** Secondary structure analysis and deconvolution of the RAGE V-domain and its Trp mutants by UV CD

V-domain mutant	β-Sheet %	Unordered %	Turn %	Helix %
WT	32.2 ± 1.2	35.6 ± 0.6	22.1 ± 0.3	10.1 ± 0.4
W51A	39.8 ± 0.6*	32.2 ± 1.8	21.4 ± 1.7	6.6 ± 0.4*
W61A	37.5 ± 1.3	31.6 ± 1.2	21.3 ± 0.8	9.6 ± 0.8
W72A	38.1 ± 0.3	32.5 ± 0.4*	20.1 ± 0.5	9.3 ± 0.4
W51A W61A	39.3 ± 1.3*	34.1 ± 0.7	21.6 ± 0.8	5.0 ± 0.3*
W51A W72A	38.4 ± 1.6	33.9 ± 0.8	22.3 ± 0.2	5.4 ± 0.5*
W61A W72A	37.0 ± 1.9	34.0 ± 2.2	23.5 ± 0.9	5.5 ± 0.6*
W51A W61A W72A	40.8 ± 1.0*	33.9 ± 0.3	21.3 ± 0.9	4.0 ± 0.4*

* Pairwise statistical analysis using the *t* test found statistically significant differences (*P*<0.05) in secondary structure composition relative to the wild-type domain.

Substitution of Trp^61^ had no significant effect on the overall secondary structure composition of the domain, while mutation of Trp^72^ increased β-sheet content at the expense of unordered residues. Mutation of Trp^51^ resulted in a significant increase in β-strand content and reduction of helical content in the single, double and triple mutants. The reduction in helical content from >10% in the wild-type domain to ∼4–5% in the mutants was statistically significant and demonstrates that the Trp residues stabilize a helical region within the V-domain. The observation that all mutants adopted a well-folded state demonstrates that the Trp residues are not required for folding of the domain.

### Fluorescence spectroscopic techniques reveal the positioning of Trp residues within the V-domain

The observed fluorescence spectra of the RAGE V-domain and its mutants are the result of the combined fluorescence contributions of all Trp residues in the system and are complicated because quantum yield, emission spectrum and fluorescence lifetime of each fluorophore is influenced by its molecular environment and neighboring fluorophores [52]. Substitution of the Trp residues with alanine simplified the analysis of fluorescence properties and thereby provided insight in solvent exposure and distances between neighboring Trp residues.

**Figure 1:**
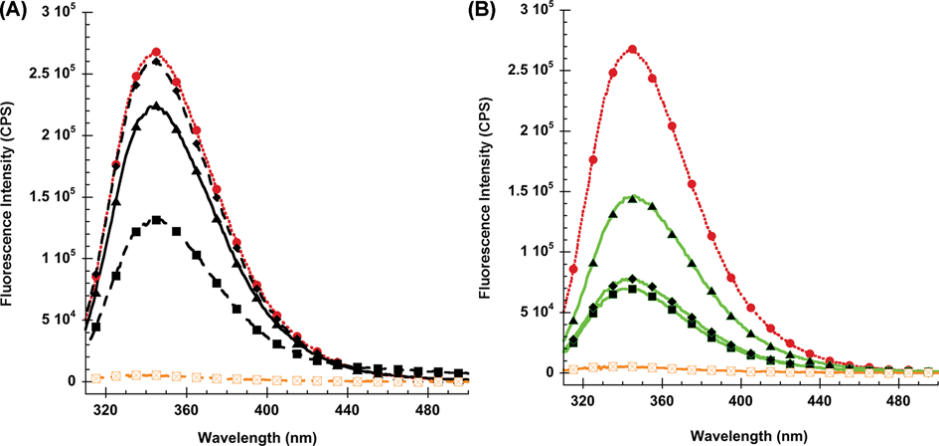
Tryptophan fluorescence of RAGE V-domains Tryptophan fluorescence emission spectra of the wild-type RAGE V-domain (solid red circles, dotted red line) and its Trp to Ala mutants. Single Trp mutants are shown in (**A**) W51A: black squares, dashed line. W61A: black diamonds, dashed line. W72A black triangles, solid line. Double Trp mutants are shown in (**B**) W51A/W61A: black squares. W51A/W72A: black triangles. W61A/W72A: black diamonds. The triple Trp mutant W51A/W61A/W72A (orange dashed line) shows minimal fluorescence signal.

The steady state fluorescence emission spectra of wild-type V-domain taken at four different excitation wavelengths (280, 285, 290 and 295 nm) showed that only the FI, but not the peak shape was affected (Supplementary Figure S3). The emission maxima (343–344 nm) did not shift with changing excitation wavelengths, demonstrating that the contribution of the two Phe and two Tyr residues in the V-domain to the overall fluorescence was negligible. The WT V-domain had an emission maximum of 344 nm with a half-peak width of 58 nm (Figure 1). This suggests that the Trp residues are predominantly exposed to a polar environment with long dipole relaxation times, typical of water molecules within the hydration shell of a protein.

The fluorescence spectra of all Trp to Ala mutants were very similar in terms of overall peak shape and emission maxima (342–345 nm). The three single Trp and the double mutants W51A/W61A and W61A/W72A experienced a slight 1–2 nm red-shift (Table 2). A more significant difference in emission maxima of 4 nm is observed for the W51A/W72A double mutant compared with the other two double mutants (W51A/W61A and W61A/W72A), suggesting that residue Trp^61^ can move into a less polar environment in the double mutants than possible for either Trp^51^ or Trp^72^.

**Table 2:** Steady state fluorescence properties of the RAGE V-domain and its Trp mutants

V-domains				V-domain:S100B complexes	
V-domain	λmax	Rel. Fl intensity	Rel. Fl intensity	λmax	Rel. Fl intensity
		High salt	High salt		
WT	344	1.00	1.00	336	1.45
W51A	346	0.41	0.46	337	1.54
W61A	345	0.87	0.65	334	1.11
W72A	345	0.60	0.61	337	1.10
W51A W61A	346	0.14	0.19	337	0.99
W51A W72A	342	0.17	0.21	336	1.30
W61A W72A	346	0.34	0.33	332	2.14

Ionic strength of the buffer (25 vs. 300 mM NaCl) had no significant effect on fluorescence emission spectra (data not shown). However, fluorescence intensities changed between low and high ionic strength conditions for some mutants, in particular the W61A mutants (Table 2). This indicates small, mutation sensitive structural changes within the V-domain. Analysis of the compiled fluorescence data demonstrates that Trp^51^ is the dominant contributor to the overall fluorescence. Trp^51^ appears to be shielded from solvent quenching and located in a protected pocket. In contrast, Trp^61^ has a relatively small contribution to overall fluorescence, most likely because the residue is located on the protein surface and subject to efficient quenching by water molecules. Trp^72^ can occupy two alternate positions. Under low ionic strength conditions, it is solvent exposed and subject to significant fluorescence quenching, while under ionic strength conditions, it becomes partially solvent protected with enhanced fluorescence emission intensity. An alternate explanation for the observed fluorescence properties is that significant fluorescence quenching occurs between Trp^51^–Trp^61^ and Trp^61^–Trp^72^ and that quenching is stronger in high salt conditions. This would indicate movement of Trp^61^ relative to the other two Trp residues of the triad in response to changes in ionic strength.

Trp fluorescence lifetimes of the wild-type and the mutant domains were measured (Table 3 and Supplementary Table S1). For the single mutants, a strong dependence of τ_m_ on the site of the Trp to Ala mutation was observed and showed that Trp^51^ contributes a long-lived fluorescence component, while Trp^72^ contributes predominately a short-lived component to the average life-times. These results are in agreement with the steady-state fluorescence experiment data that show that Trp^51^ is sequestered from solvent, while Trp^72^ is predominantly solvent accessible. All double mutants show short life-times (τ_m_ 3.3–3.6 ns), demonstrating that the remaining single Trp residues are not effectively solvent shielded in these mutants.

**Table 3:** Fluorescence lifetimes of the RAGE V-domains and their S100B complexes

	τ_μ_ (ns)	τ_m_ (ns)	
Sample	V-domain	V-domain:S100B	Δτ_m_ (ns)
WT	5.08 ± 0.30	4.25 ± 0.08	−0.83*
W51A	3.99 ± 0.04	4.14 ± 0.15	+0.15
W61A	4.95 ± 0.17	4.15 ± 0.07	−0.80*
W72A	5.47 ± 0.09	4.57 ± 0.01	−1.00*
W51A W61A	3.46 ± 0.03	3.41 ± 0.10	−0.05
W51A W72A	3.34 ± 0.05	3.55 ± 0.32	+0.21
W61A W72A	3.61 ± 0.02	4.22 ± 0.16	+0.61*

* Statistical significance based on unpaired *t* test (0<0.05). The unfolded state of all domains exhibited a τ_m_ 3.1–3.3 ns.

**Figure 2:**
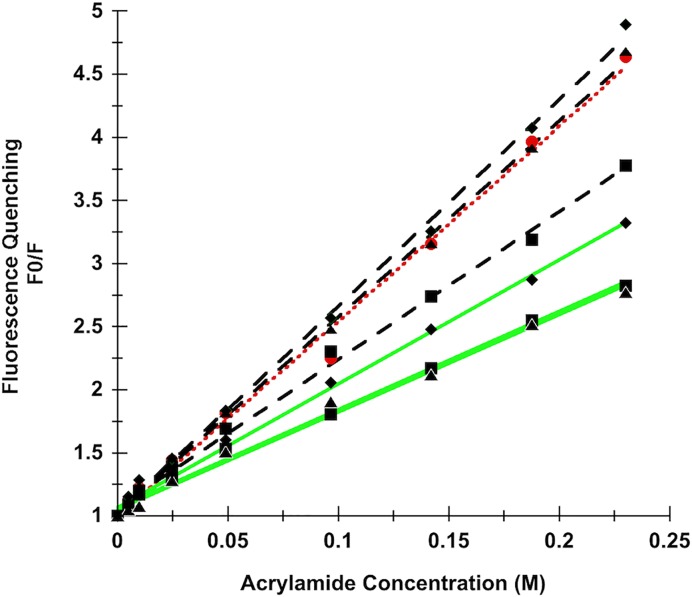
Collision quenching of Trp fluorescence of RAGE V-domains Stern–Volmer plots of acrylamide quenching of Trp fluorescence of the RAGE V-domain and its Trp mutants. The wild-type V-domain is shown by solid red circles and a dotted red line. The single Trp mutants are shown by black, dashed lines (W51A squares, W61A diamonds and W72A triangles), the double Trp mutants are shown by solid green lines (W51/W61 squares, W51A/W72A triangles and W61A/W72A diamonds).

Collision fluorescence quenching was used to further corroborate the solvent accessibility of each Trp residue determined from steady state and time resolved fluorescence experiments. Acrylamide quenching titrations for the wild-type and all V-domain mutants were performed and Stern–Volmer quenching constants *K*_sv_ were calculated (Figure 2) and used to estimate the fraction of ‘quencher inaccessible’ fluorescence (Table 4).

**Table 4:** Acrylamide fluorescence quenching of RAGE V-domain tryptophan mutants

V-domain mutant	*K*_sv_ (M^−1^)	Quenching rate constant k_q_ M^−1^.s^−1^ (acrylamide)	Fractional quenching accessibility
WT	15.52 ± 0.29	3.05 ± 0.12 × 10^9^	0.85 ± 0.04
W51A	12.16 ± 0.22	3.06 ± 0.15 × 10^9^	0.90 ± 0.01
W61A	16.57 ± 0.20	3.35 ± 0.14 × 10^9^	0.80 ± 0.05
W72A	15.71 ± 0.16	2.87 ± 0.11 × 10^9^	0.78 ± 0.02
W51A W61A	8.91 ± 0.19	2.37 ± 0.14 × 10^9^	0.70 ± 0.07
W51A W72A	8.04 ± 0.21	2.40 ± 0.14 × 10^9^	0.76 ± 0.03
W61A W72A	10.25 ± 0.18	2.83 ± 0.16 × 10^9^	0.74 ± 0.02

Data of the quenching experiments generally showed high accessibility of the Trp residues, indicating significant solvent exposure. Data for the double mutants suggest that Trp^51^ (mutant W61A/W72A) is less accessible to the quencher than Trp^61^ or Trp^72^. A similar conclusion can be drawn for the single Trp V-domain mutants. In addition, Trp^61^ seems to be slightly more accessible to the quencher than residue Trp^72^. These results are in agreement with our previous data demonstrating that Trp^51^ is located in the protein interior, whereas Trp^61^ and Trp^72^ are mostly solvent exposed.

### Sypro-orange binding and differential scanning fluorimetry confirm the positioning of Trp side chains in the V-domain

**Figure 3:**
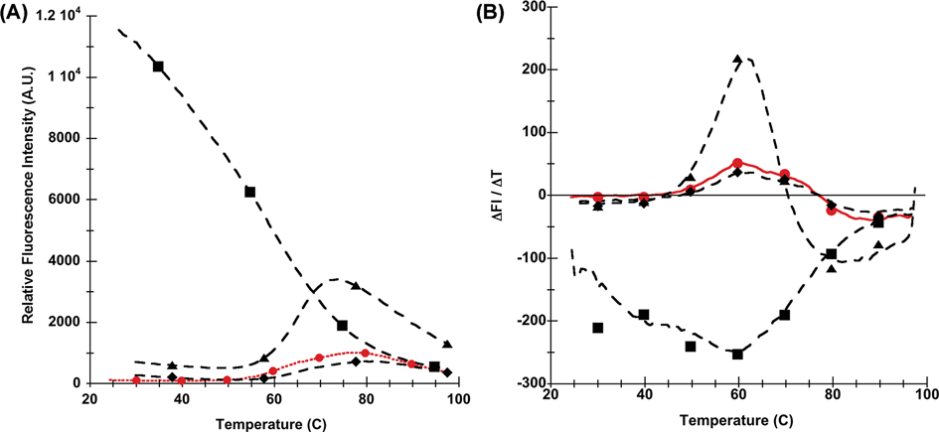
Thermofluor assay of RAGE V-domains Thermofluor assay profiles of the RAGE V-domain and its single Trp mutants. SYPRO Orange was used as fluorescence probe. Shown in (**A**) are the changes in SYPRO Orange FI as a function of temperature (T). (**B**) shows the differential changes in fluorescence ΔFI/ΔT as a function of temperature (T). The WT (red) and the single Trp mutants are shown in the top panels (black, dashed lines, W51A squares, W61A diamonds and W72A triangles). Thermofluor assay traces for the double and triple mutants are shown in Supplementary Figure S4.

The Sypro-orange dye greatly increases its FI when stripped of bulk water molecules and placed into a more hydrophobic environment. These properties of SYPRO Orange are used to measure the exposure of hydrophobic areas during temperature-induced protein unfolding (Thermofluor Assay) [39,53]. Thermofluor assay traces for the wild-type V-domain and the three single mutants are shown in Figure 3. A very strong fluorescence signal at low temperature is observed for the W51A mutant, but not for the wild-type, W61A or W72A mutants. The observed high Sypro-orange FI for the W51A mutant at low temperature could be explained by Sypro-orange binding to a hydrophobic cavity in the interior of the V-domain. It has previously been shown that protein cavities engineered by the substitution of large amino acid side chains with small side chains can be accessible to soluble ligand molecules [54,55]. Alternatively, the strong Sypro-orange FI could result from binding to a hydrophobic surface on the W51A mutant. This would suggest a structural weakening of the W51A mutant and partial transition to a molten globule state [56].

For proteins with well defined thermal melting transitions, Sypro-orange fluorescence increases as the protein transitions to a molten globule state where the Sypro-orange dye binds to hydrophobic sites formed due to hydrophobic collapse of the protein fold [53]. This behavior is seen for WT, W61A and particularly the W72A mutant, with well-defined inflection points in plots of fluorescence intensities (FI) versus temperature (T) (Figure 3A) and corresponding maxima in FI/ΔT versus T plots (Figure 3B). The overall shape of the thermofluor data indicate that the V-domain and its mutants undergo a gradual melting with a midpoint ∼60°C (see Supplementary Figure S4 for thermofluor assay results for double mutants). This is in agreement with data by Dattilo et al. [50] who reported slow thermal unfolding based on differential scanning calorimetry experiments.

### Trp residues are not required for S100B binding to the V-domain

In order to obtain a binding measurement independent of protein conformation, binding of the RAGE V-domain and its mutants to FITC-labeled S100B was measured by fluorescence polarization titration (Table 5 and Supplementary Figure S5).

**Table 5:** Binding affinity of S100B to the RAGE V-domain and its mutants

V-domain	S100B:V-domain
*K*_D_ (μM)	
WT	2.7 ± 0.3
W51A	0.48 ± 0.06
W61A	0.87 ± 0.04
W72A	1.12 ± 1.01
W51A W61A	0.66 ± 0.12
W51A W72A	0.46 ± 0.32
W61A W72A	0.37 ± 0.09

S100B binds wild-type V-domain with an apparent binding affinity of *K*_d_ = 2.7 μM, which is slightly weaker than previously reported binding affinities measured by SPR (*K*_d_ = 0.5 μM) [1]. The Trp to Ala mutants showed slightly tighter binding with *K*_d_’s between 0.4 and 1.1 μM (Table 5).

### Residues Trp^62^ and Trp^72^ are part of the protein–protein interface in the V-domain:S100B complex

The detailed fluorescence spectroscopic characterization of the RAGE V-domain and its mutants (Tables 2 and 3) provided the foundation for the similar characterization of the role of the three tryptophan residues in the V-domain:S100B complex. S100B does not contain Trp residues and the observed changes in Trp fluorescence can be exclusively attributed to structural changes occurring within the environment of Trp residues of the V-domain. Binding of S100B to the V-domain and its mutants blue shifted the Trp fluorescence emission maxima to shorter wavelengths by 6–14 nm and increased the fluorescence emission intensities up to 2.1-fold (Table 2). In the V-domain:S100B complex, the Trp residues are therefore in a more hydrophobic environment and better shielded from solvent water molecules than in the non-complexed V-domain. Analysis of the data for single and double mutants suggests that Trp^61^ and Trp^72^ become partially shielded from solvent molecules in the V-domain:S100B complex. The shielding effect is only slightly more pronounced for residue Trp72 than for Trp61.

The data for Trp^51^, which is located in the interior of the V-domain, also show that binding of S100B causes a decrease of polarizability of the environment of the indole ring, as well as a decrease in fluorescence quenching. S100B binding, while not directly interacting with Trp^51^, appears to alter the inner side chain packing of the V-domain slightly, resulting in changes in the local environment of Trp^51^.

Structural changes to the interior amino acid packing of the V-domain near Trp^51^ may be required to facilitate the binding of S100B. This is further evidence that the V-domain makes use of structural plasticity to bind S100B.

Changes in measured fluorescence lifetimes upon addition of S100B also reflect changes in the physico-chemical environment of the Trp fluorophores (Table 3). The amplitude weighted average fluorescence lifetime decreased significantly by ∼0.8 ns when S100B bound to the wild-type V-domain, clearly reflecting the interaction between the two proteins. Similar changes in fluorescence lifetime were seen for W61A (0.8 ns) and W72A (1.0 ns) mutants. However, the W51A mutant experienced no statistically significant change in fluorescence lifetime. The double Trp mutants showed no significant changes in fluorescence lifetimes, whereas W61A/W72A did show an increase in fluorescence lifetime by 0.6 ns. However, our detailed interpretation regarding the local environment of individual Trp residues in the V-domain mutant:S100B complexes is uncertain because of the complexity of three Trp residues in the fluorescence system and the multiple factors that affect fluorescence lifetimes.

### X-ray crystallographic analysis of the S100B:W72 peptide complex

A crystallographic approach was explored to further investigate the RAGE V-domain Trp^72^:S100B interaction at the atomic level. The RAGE-derived W72 peptide, corresponding to RAGE residues 65–79, was successfully crystallized in complex with calcium-loaded S100B. Data collection and refinement statistics are reported in Table 6. The crystal structure was refined with one W72 peptide (SPQGG**GPWDSVARVL**, residues modeled in electron density in bold and number as residues 1–10 in the PDB deposited structure 5D7F) bound to an S100B dimer. Each S100B monomer exhibits an all α-fold consisting of four helices arranged in two EF-hand Ca^2+^-binding motifs. The S100B homodimer interface comprises a symmetric X-type four-helix bundle. Comparison of the apo-Ca^2+^-bound S100B [57] with the S100B:W72 peptide complex reveals that, although the overall fold is conserved, there are variations in the C-termini and the hinge region between helices 2 and 3.

**Table 6:** X-ray diffraction data-reduction and refinement statistics

Data collection	
Beamline	NE-CAT 24-ID-C
Wavelength (Å)	0.9792
Space group	*P2_1_*
Unit-cell parameters (Å, °)	*a* = 35.1, *b* = 59.8, *c* = 47.6
	α = γ = 90, β = 111.7
Molecules in asymmetric unit	3
Resolution range (Å)	44.2–1.17
Total observations	58236 (2949)
Unique observations	43997 (1529)
Multiplicity	3.2 (1.7)
Completeness (%)	97.9 (98.2)
R_merge_ (%)	4.1 (82.8)
Average I/σI	23.2 (4.42)
Data-processing program	RAPD (XDS)
Refinement	
Refinement program	PHENIX
Resolution range (Å)	44.2–1.30 (1.35–1.30)
R_work_ (%)	15.7
R_free_ (%)	18.3
RMSD stereochemistry	
Bond lengths (Å)	0.014
Bond angles (°)	1.63
Number of atoms	3628
S100B	1609
W72 peptide	154
Waters	214
Ca^2+^	4
Average *B* (Å^2^)	
S100B	20.4
W72 peptide	24.4
Waters	30.7
Ca^2+^	16.5
Ramachandran plot (%)	
Preferred	96
Allowed	4
Outliers	0
PDB code	5D7F

Values in parentheses pertain to the highest resolution shell.

$R_{merge}=\sum_{hkl}\sum_{i}\left|I_{i}(hkl)-\langle I(hkl)\rangle \right|/\sum_{hkl}\sum_{i}I_{i}(hkl)$

**Figure 4:**
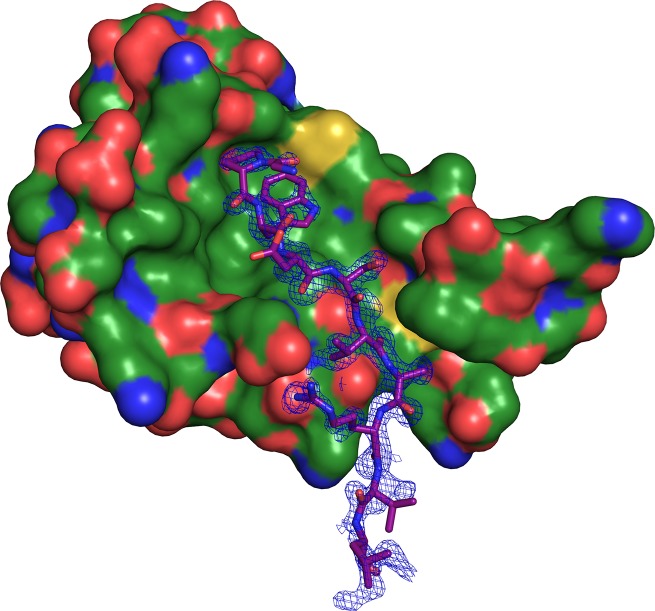
Binding orientation of peptide W72 on the S100B surface Positioning of the W72 peptide on the surface of S100B. The final refined 2F_o_–F_c_ electrondensity (blue mesh) contoured at 1σ above the mean of the composite OMIT map.

The W72 peptide binds the hydrophobic groove on the surface of S100B, positioned between helices 3 and 4 and the hinge region between helices 2 and 3, similar to other S100B:peptide complexes [33,58–60]. The W72 peptide adopts a random coil conformation, with the N-terminus positioned for extension beyond the hydrophobic groove, although these residues are not resolved in the electron density (Figure 4).

As observed in other S100B:peptide complex structures [33,58], the association of the W72 peptide with S100B involves hydrophobic interactions and hydrogen bonds. Five hydrogen bonds are observed in the S100B:W72 peptide complex. S100B Glu^46^ is involved in three hydrogen bonds; it interacts with Arg^8^ (corresponding to Arg^77^ of the RAGE V-domain) and Asp^4^ (corresponding to Asp^73^ of the RAGE V-domain) on the W72 peptide. The peptide Arg^8^ residue also forms a hydrogen bond with the His^43^ residue of S100B. Val^6^ of the peptide (corresponding to Val^75^ of the V-domain) is involved in a hydrogen bond with Phe^44^ of S100B. Additionally, the interaction between the W72 peptide is stabilized by a bidentate salt bridge between Arg^8^ of the W72 peptide and S100B Glu^46^. Interestingly, salt bridges are not involved in the formation of the S100B:W61 or the S100B:TRTK12 peptide complexes [33,58].

**Figure 5:**
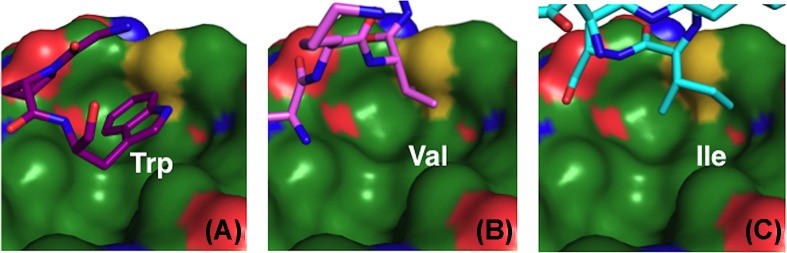
Placement of hydrophobic peptide residues on the S100B surface The hydrophobic binding side on the S100B surface that is occupied by Trp in the S100B:W72 structure (**A**) is filled by Val in the S100B:W61 complex (**B**) and by Ile in the S100B:TRTK12 structure (**C**).

PISA webserver [49] analysis of the interface between S100B and the W72 peptide indicated a total buried surface area of 537.8 Å^2^, which is 34.4% of the total peptide surface area. Notably, Pro^2^ and Trp^3^ of the W72 peptide (corresponding to Pro^71^ and Trp^72^ of the V-domain) are 80.4 and 85.4% buried (102.17 and 164.43 Å^2^, respectively). The indole ring of Trp^3^ is located inside a hydrophobic surface depression lined by S100B residues Ile^36^, Leu^44^, Ile^47^, Val^56^, Leu^60^, Phe^76^, Met^79^ and Val^80^. Trp^3^ is further shielded from solvent by peptide residue Gly^70^. Several S100B:peptide complexes solved by X-ray crystallography incorporate a Trp-containing peptide and the S100B:peptide complexes display variability in the positioning of the Trp within the S100B hydrophobic groove. Each Trp indole ring occupies a different cavity. For the S100B:TRTK12 complex, the site occupied by Trp for TRTK12 is populated by Ala^60^ of the W61 peptide [33]. Similarly, the hydrophobic pocket that positions Trp^3^ of the W72 peptide (Figure 5A) is occupied by Val^7^ in the W61 peptide (Figure 5B) and Ile^10^ in the TRTK12 peptide (Figure 5C). The Trp in the S100B:W72 peptide complex exhibits the greatest buried surface area at 164.4 Å^2^, compared with 65.9 Å^2^ for Trp^61^ and 141.2 Å^2^ for Trp^7^ in the S100B:W61 peptide and S100B:TRTK12 complexes, respectively (Supplementary Table S3).

**Figure 6:**
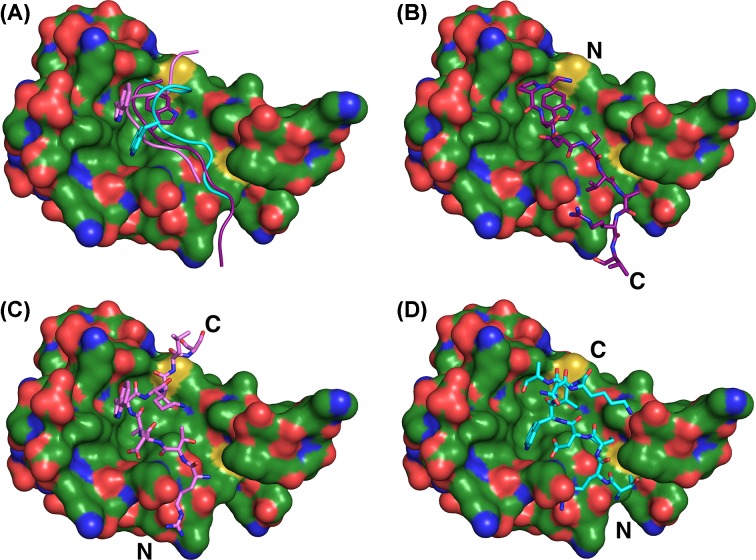
Positioning of the W72, W61 and TRTK12 peptides on the S100B surface Comparison of the relative orientation of the W72-peptide (**B**), the W61-peptide (**C**) and the TRTK-12 peptide (**D**) on the S100B surface. The N-and C-termini of the peptides are indicated. The positioning of Trp residues in hydrophobic surface areas is shown in (**A**).

The orientation of the N- and C-termini for the W72 peptide is reversed relative to W61 and TRTK12 peptides (Figure 6). Further, the W72 peptide has a four residue extension beyond the hydrophobic groove. In the S100B:W72 peptide structure, the C-terminus extends beyond the hinge region, with the N-terminus positioned for extension beyond helix 3. Both the S100B:W61 peptide and S100B:TRTK12 structures orient the C-termini near helix 3, with the N-termini positioned near the hinge region (Figure 6).

Overall, nearly the same residues of S100B are buried in the interaction with all three peptides. The amount of total buried surface area for the three complexes is fairly close (1010.4 Å^2^ for the W72 peptide, 933.7 Å^2^ for the W61 peptide and 1084.3 Å^2^ for TRTK-12). Significant differences were observed in the buried surface area of certain residues. His^43^ had a very large buried surface area of 58.86 Å^2^ (34.2%) in the S100B:W72 peptide structure and buried surface area of 32.24 and 32.71 Å^2^ in the TRTK-12 and W61 peptide structures, respectively. The buried surface area of the Glu^46^ residue was also significantly higher in the W72 peptide when compared with TRTK-12. Met^80^, Ala^84^, Glu^87^ and Phe^88^ residues also had significantly different buried surface areas between the TRTK-12 and the W72 peptide structures (Supplementary Table S2).

## Discussion and conclusion

The present study is the first to use site-directed mutagenesis to interrogate the role of three specific tryptophan residues for the structural stability of the RAGE V-domain and its ligand binding properties. The detailed characterization of the wild-type V-domain and of seven engineered Trp-Ala mutants, using CD, multiple steady-state and time-resolved florescence spectrometric methods and direct protein binding studies resulted in a model for the structural dynamic behavior of the V-domain. S100B was chosen as a model ligand for RAGE and conclusions drawn here regarding the structural plasticity of the V-domain upon interaction with S100B are also relevant to other RAGE ligands.

Because RAGE is activated by binding structurally diverse ligands, it has long been assumed that some degree of structural plasticity is required for ligand binding. Some classes of RAGE ligands possess more structural plasticity than others. For example, AGE compounds are heterogeneously modified and partially destabilized in their secondary and tertiary structures, thus binding of AGE to RAGE can be speculated to be achieved by structural accommodation within the AGE ligand. S100 proteins on the other side are structurally fairly rigid, in particular in their Ca^2+^-bound state. This raises the question of which part of the RAGE V-domain undergoes structural changes when binding S100B? NMR studies have suggested that the RAGE–S100B interaction is predominantly electrostatic. However, that fails to explain the strong calcium-dependence of RAGE-S100B binding and the observation that many S100B–target interactions make use of hydrophobic anchor residues. The previously identified S100B consensus binding sequence (K/R(L/I)XWXXIL) contains a central Trp residue that functions as an anchor residue to a hydrophobic depression on the S100B surface in several S100B:peptide complexes [26]. The sequences surrounding Trp^61^ and Trp^72^ are similar to the consensus sequence, suggesting that either Trp residue could function as an anchor residue.

A systematic, side directed Trp to Ala mutational approach and fluorescence spectrometric studies provided insight in the local environment of each Trp residue and how that environment changes when one of the other Trp residues is mutated or when S100B is present. Circular spectroscopic data provided information on the overall secondary folding state of the domain in general and a short helical region involving residues 70–76 in particular.

The three Trp residues (Trp^51^, Trp^61^ and Trp^72^) form a Trp triad, a system of Trp residues that are close to each other in the folded domain and that structurally and spectroscopically communicate with each other. Thus, mutating one residue effects the local environment and thus the spectroscopic properties of the other Trp residues. A comprehensive analysis of the experimental data revealed that Trp^51^ is predominantly solvent shielded and located in the interior of the V-domain. This is in agreement with published structural data that show that Trp^51^ embedded in the outer strand of a β-sheet with the indole side chain oriented toward the protein interior. In all Trp mutants and in the S100B complex, the local environment of Trp^51^ does not appear to change much. Trp^61^ is predominantly surface exposed and embedded in a partially unstructured region connecting Trp^51^ and Trp^72^. Trp^72^ is also mostly solvent exposed, but can adopt a partially solvent shielded positioning as well. Studies have shown that Trp^72^ is part of a region (residues 70–76) that can adopt a helical conformation. This helical segment is stabilized by the Trp triad and mutation of any Trp residues results in a shortening or partial unfolding of the helix by ∼50%. As a consequence, structural plasticity increases, allowing the Trp residues to alter their relative positioning in the domain.

Changes in Trp fluorescence clearly showed that S100B binding altered the local environment of the three Trp residues and shielded in particular residues Trp^61^ and Trp^72^ from solvent exposure. This is likely due to direct binding of the Trp residues to the S100B surface. However, the possibility that S100B binds to a different region of the V-domain and subsequently induces the formation of V-domain dimers cannot be completely dismissed. Somewhat unexpectedly, S100B binding was not lost by Trp to Ala mutations, and even the triple mutant retained high S100B-binding affinity. This observation is interpreted as reflecting a shift between the two principle types of binding interactions in the V-domain:S100B complex: hydrophobic interactions (e.g. anchor residues) and electrostatic interactions as result of structural plasticity in the V-domain.

To gain further insight into the possible binding modes between the V-domain and S100B on the atomic level, two crystal structures of RAGE-derived peptides containing either Trp^61^ (peptide W61 [33,58]) or Trp^72^ (peptide W72, this paper) bound to S100B have been solved. The structures suggest two alternate binding modes between RAGE and S100B. The most significant differences between the two binding modes are that, (i) the two peptides are oriented in opposing directions on the S100B surface and (ii) the Trp residues do not occupy the same hydrophobic anchor pocket of S100B. In the W72 peptide, Trp^72^ is a hydrophobic anchor residue, while in the W61 structure, Val^63^ acts as a hydrophobic anchor residue. The crystallographic data thus support the concept that S100B can bind ligands in multiple orientations on its surface and suggests that the V-domain could utilize multiple binding modes to interact with S100B. The concept of multiple binding modes involving the Trp^51^–Trp^72^ region of the V-domain and S100B is also supported by the Trp fluorescence data presented here. Further, our observation that the triple Trp to Ala mutant also binds to S100B is in line with the binding of protein without a Trp anchor residue, as it was observed in the S100B:RSK-1 peptide complex [61].

Taken all the available information together, it becomes clear that the RAGE V-domain has not evolved for high-affinity binding of the S100B ligand. Instead, the Trp triad plays an important role in modulating the structural plasticity of the V-domain and facilitating binding of S100B via at least two distinct modes. The existence of multiple binding modes between the V-domain and S100B may also explain the difficulties to obtain diffraction quality crystals of the complex for X-ray analysis.

**Figure 7:**
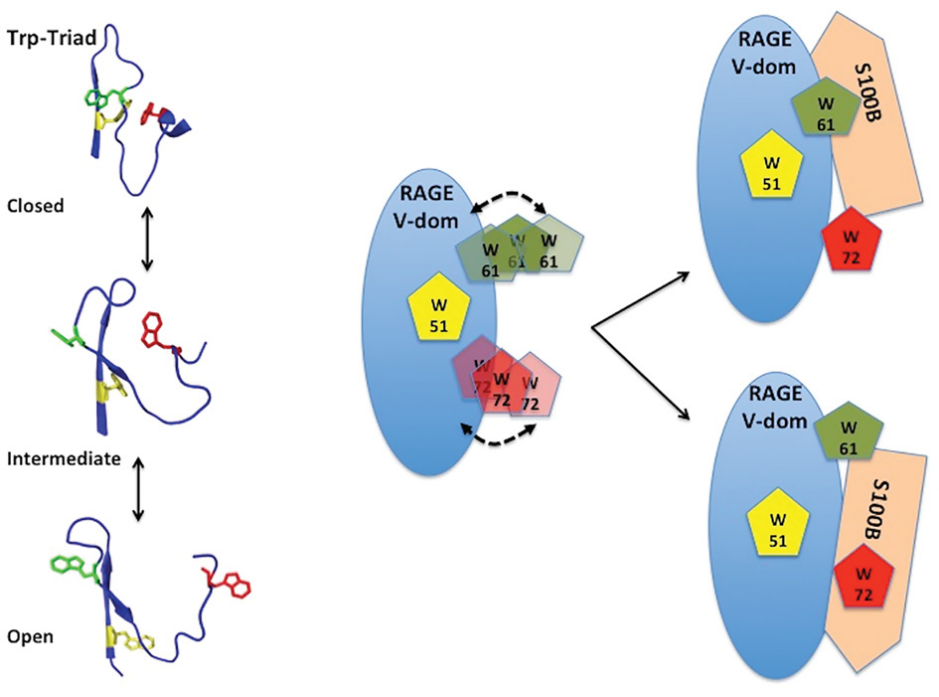
Structural plasticity of the RAGE V-domain Trp triad suggests multiple binding modes for S100B Examples of the open, closed and intermediate conformations of the Trp triad of the RAGE V-domain. The shown conformations are based on PDB entries 2E5E (closed), 4P2Y (intermediate) and 2L7U (open). Residues 49–74 are shown as cartoon, Trp^51^ (yellow), Trp^61^ (green) and Trp^72^ (red) are shown as stick models. Residues Trp^61^ and Trp^72^ assume multiple orientations on the RAGE surface, which allows for the binding of S100B in at least two distinct binding modes.

A review of available NMR and X-ray structures is in agreement with the conclusions reported here and demonstrates multiple conformational states for the Trp triad. Distances between the Trp residues of the triad vary significantly and can be grouped into three classes: a closed triad, where distances between the Trp β-carbons are between 6 and 8 Å (Trp^51^–Trp^61^: 7–8 Å, Trp^51^–Trp^72^: 8 Å, Trp^61^–Trp^72^: ∼8 Å; based on PDB 2E5E); an open triad, where distances are much greater (Trp^51^–Trp^61^: 10–12 Å, Trp^51^–Trp^72^: 14–18 Å, Trp^61^–Trp^72^: 19–23 Å, based PDB 4P2Y); and an intermediate Trp triad arrangement (Trp^51^–Trp^61^: 10–11 Å, Trp^51^–Trp^72^: 10 Å, Trp^61^–Trp^72^: 13 Å; based on PDB 2L7U) (Figure 7). It is likely that the region including the residues of the Trp triad undergoes conformational changes upon interaction with a potential ligand and that structural plasticity is used to sample conformations that facilitate binding of a specific ligand.

It thus becomes apparent that a lock-and-key binding mode for the RAGE:S100B complex is not an appropriate model for the complex. The actual details of the structure and stoichiometry of the RAGE:S100B complex under physiological conditions remains unknown and may in fact vary depending on neighboring membrane proteins and peptidoglycans. Besides those remaining unknowns, the results of the present study propose that residues Trp^61^ and Trp^72^ of the V-domain could both mediate the binding of S100B to RAGE. This suggests that this region of the V-domain may be a suitable target region for ligand-specific RAGE antagonist or possibly agonists as well. This could be achieved by developing monoclonal antibodies directed against this epitope or by identifying small organic molecules that restrict the structural plasticity of the V-domain.

## Accession codes

Uniprot protein accession codes RAGE Q15109, S100B P04271. The atomic coordinates and structure factors reported in the present paper have been deposited in the Protein Data Bank [5D7F]. PDB entries used are listed in Table 6.

## Supplementary Material

Supplementary Figures S1-S5 and Tables S1-S3

## Abbreviations

AGE: advanced glycation end product
CD: circular dichroism
DAMP: damage-associated molecular pattern
FI: fluorescence intensity
FITC: fluorescein isothiocyanate
GuHCl: guanidinium chloride
IPTG: isopropyl-beta-D-thiogalactoside
RAGE: receptor for advanced glycation end product
SPR: surface plasmon resonance
Trp^51^, Trp^61^ and Trp^72^: refer to tryptophan residues in positions 51, 61 and 72 of the RAGE V-domain
WT: wild type
W61 and W72: refer to synthetic peptides corresponding to RAGE residues 54-68 (NTGRTEAB W KVLSPQG) and 65-79 (SPQGGGP W DSVARVL)
W51A, W61A and W72A: refer to substitution of the indicated tryptophan residues by alanine in the RAGE V-domain
