# Supplementary material for: The Trp triad within the V-domain of the receptor for advanced glycation end products modulates folding, stability and ligand binding
Source: Biosci Rep. 2020 Jan 31;40(1):BSR20193360. doi: 10.1042/BSR20193360 (PMC6997106; doi:10.1042/BSR20193360)
Supplement: Supplementary Figures S1-S5 and Tables S1-S3 [file BSR-2019-3360_supp.pdf]

Supplemental Information:

The Trp-Triad within the V-Domain of the Receptor for Advanced Glycation Endproducts (RAGE) Modulates Folding, Stability, and Ligand Binding

Venkata S.K. Indurthi, Jaime L. Jensen, Estelle Leclerc, Sangita Sinha, Christopher L. Colbert, Stefan W. Vetter

Expression and purification of RAGE V-domain proteins

Description CD measurements

Table S1: Fluorescence life times

Table S2: Burried peptide surface areas

Figure S1: SDS-PAGE of purified WT V-domain

Figure S2: CD traces of V-domain proteins

Figure S3: Fluorescence emission spectra

Figure S4: Thermofluor assay traces for double and triple Trp to Ala V-domain mutants

Figure S5: Representative V-domain – S100B fluorescence polarization titration fits

Expression and Purification of RAGE V-domain proteins

Proteins were expressed in the disulfide isomerase (DsbC) expressing *E.coli* strain Shuffle T7 Express (New England Biolabs, Cat# C3029). LB media (10g/L tryptone, 5 g/L yeast extract, 10 g/L NaCl, pH 7.4).

Cells were grown in multiple 2,000 ml, no-baffled glass Erlenmeyer flasks with 400 ml LB at 37°C in a shaking incubator. Protein expression was induced at an OD_{600} of 0.6 and the temperature was then reduced to 30°C and cells were harvested after 4 hours by centrifugation. The cell pellet was resuspended in 50 mM Tris, 20 mM imidazole, 300 mM NaCl, pH 8.0 and frozen at -20°C.

Cell were thawed on ice and sonicated (Misonic XL-2000, equipped with a P4 tip) for 5-10 cycles of 20 seconds with intermittent chilling on ice until the viscosity of the sonicated cell slurry was comparable to water when dripped from a Pasteur pipette. This simple drip-test was used to ensure sheering of DNA. Cell debris was separated by centrifugation (17,000 x g) at 4°C for 20 min. The clarified supernatant was passed through a 0.45μm syringe filter and loaded onto a His-Trap HP column (GE Healthcare). A Biorad Biologic DuoFlow chromatography system was used for all chromatography steps. After washing the column with 50 mM Tris, 20 mM imidazole, 300 mM NaCl, pH 8.0, the bound protein was eluted with 50 mM Tris, 200 mM imidazole, 300 mM NaCl, pH 8.0 and fractions were collected. Protein elution was monitored by an inline UV absorbance detector. Protein containing fractions were further identified by visual Bradford protein assay using 20 μl from each fraction and 200 μl Bradford reagent (Biorad). Fractions containing the highest protein amounts were pooled and diluted 1:1 with 10 mM Na-acetate buffer, pH 5.5. The diluted protein solution was loaded onto a pre-equilibrated (10 mM Na-acetate, pH 5.5) HiTrap SP FF column (GE Healthcare) and eluted with a linear salt gradient (10 mM Na-acetate, 1 M NaCl, pH 5.5). Protein elution was monitored by inline UV monitoring and Bradford assay on single fractions. This second chromatography step removed oligonucleic acid fragments that were co-eluted with V-domain during the His-tag affinity purification step. Pooled fractions were concentrated using an Amicon ultrafiltration device with 3kDa MWCO membranes. Protein purity was confirmed using SDS-PAGE, followed by Coomassie blue staining.

CD Measurements and analysis

CD-measurements were performed on a JascoJ815 spectropolarimeter equipped with a PFD-425 Peltier cell holder. A 1 mm path length quarz cuvette was used and protein concentrations of 25 μM in 50 mM Tris, 150 mM NaCl, pH 7.0 were used for all measurements. The

temperature of the cell holder was set to 20.0°C. Spectral data were continuously collected between 180-260 nm in 0.5 nm increments, with a digital integration time (D.I.T.) of 8 seconds, a band with of 4 nm and a scanning speed of 10 nm/min. Ten accumulations were averaged by the instrument software. For each V-domain protein 5 independent samples measured (n=5) and each was deconvoluted using CONTIN in the DichroWeb software. Differences in the compositions of the secondary compositions were then statistically analyzed by pairwise student t-test.

Supplemental Figure S1: Coomassie stained SDS PAGE of purified RAGE wild type V-domain. The Thermofisher prestained protein maker page ruler 10-180 kDa prod #26616 is shown in the left lane.

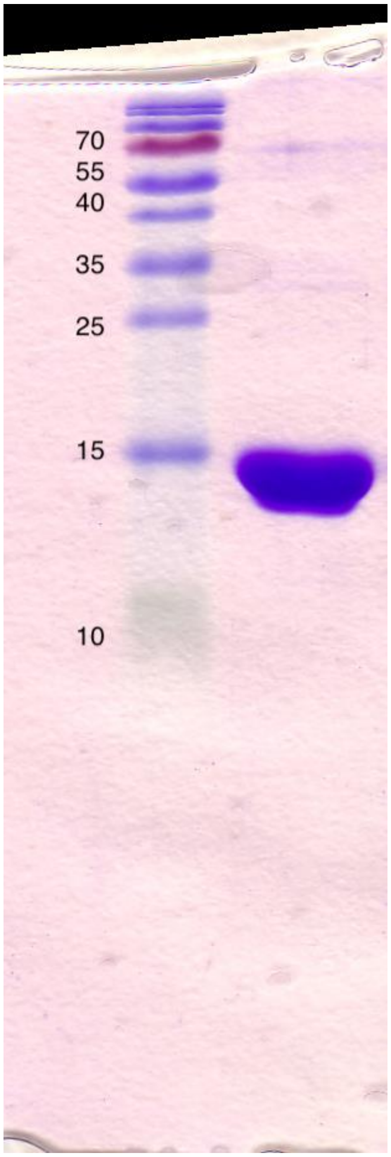

Supplemental Figure S2: Representative CD spectra of the WT-V-domain and its mutants: WT (dotted red), W51A (black squares, black dashed line), W61A (black diamonds, black dashed line), W72A (black triangles, black dashed line), W51A/W61A (black squares, solid green line), W51A/W72A (black squares, solid green line), W61A/W72A (black triangles, solid green line), W51A/W61A/W72A (open squares, dashed orange line)

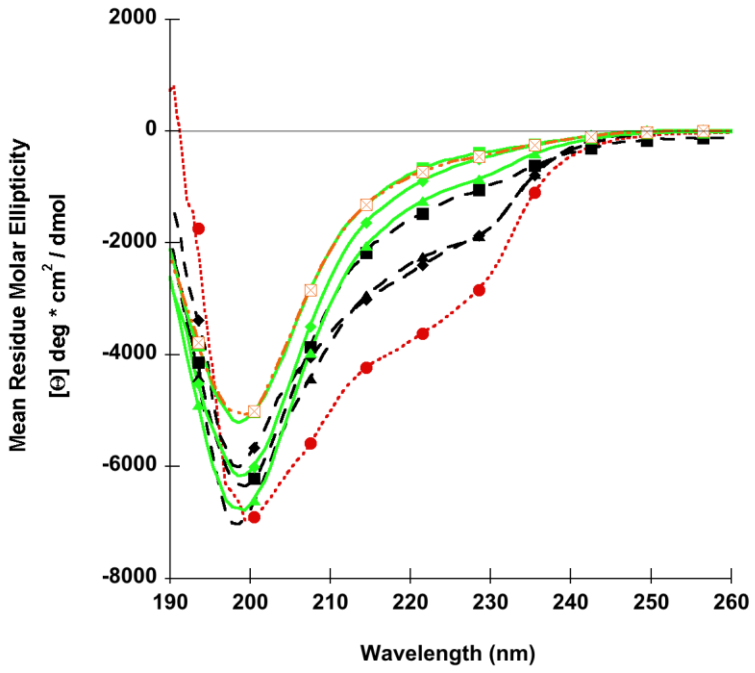

Supplemental Figure S3: WT V-domain fluorescence emission spectra for different excitation wavelengths

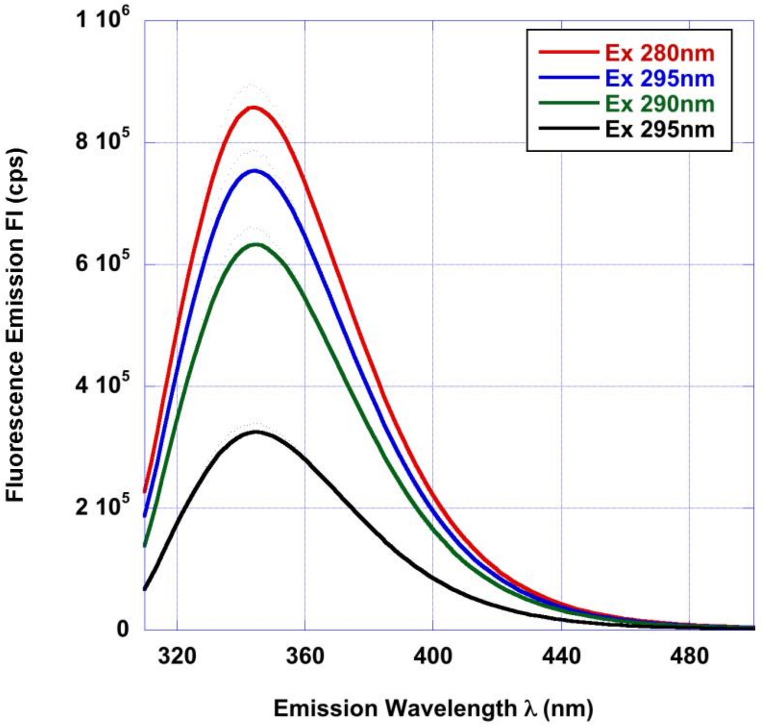

Differential fluorescence intensity ΔFI/λ versus emission wavelength λ

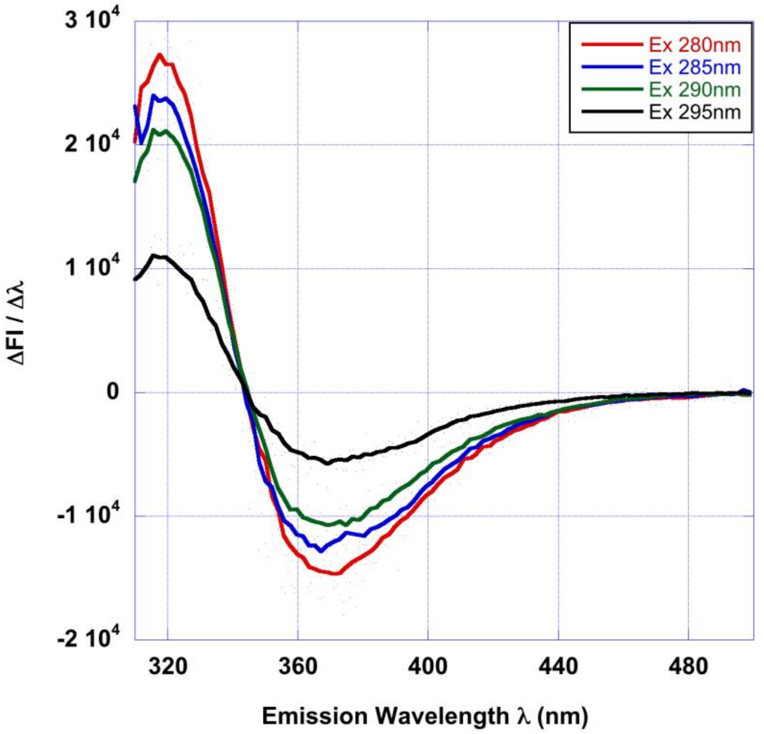

Supplemental Figure S4: Thermofluor assay traces for double and triple Trp to Ala V-domain mutants

Trp mutants: W51A/W61: black squares, W51A/W72A: black diamonds, W61A/W72A: black triangles; the triple mutant (W51A/W61A/W72A) is shown in orange.

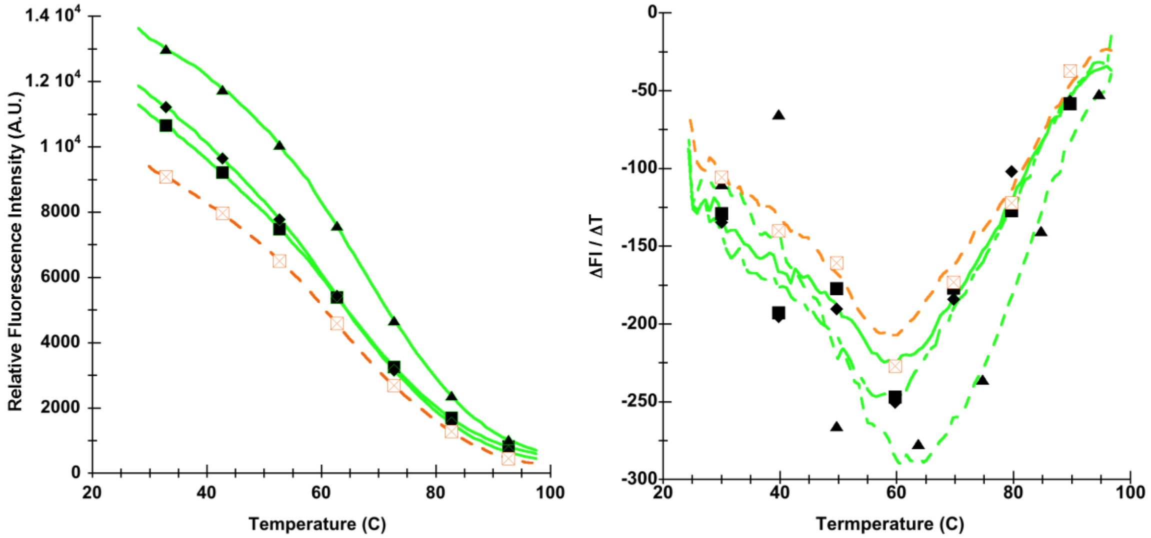

Supplemental Figure S5: V-dom: Representative S100B - V-domain fluorescence polarization titration fits

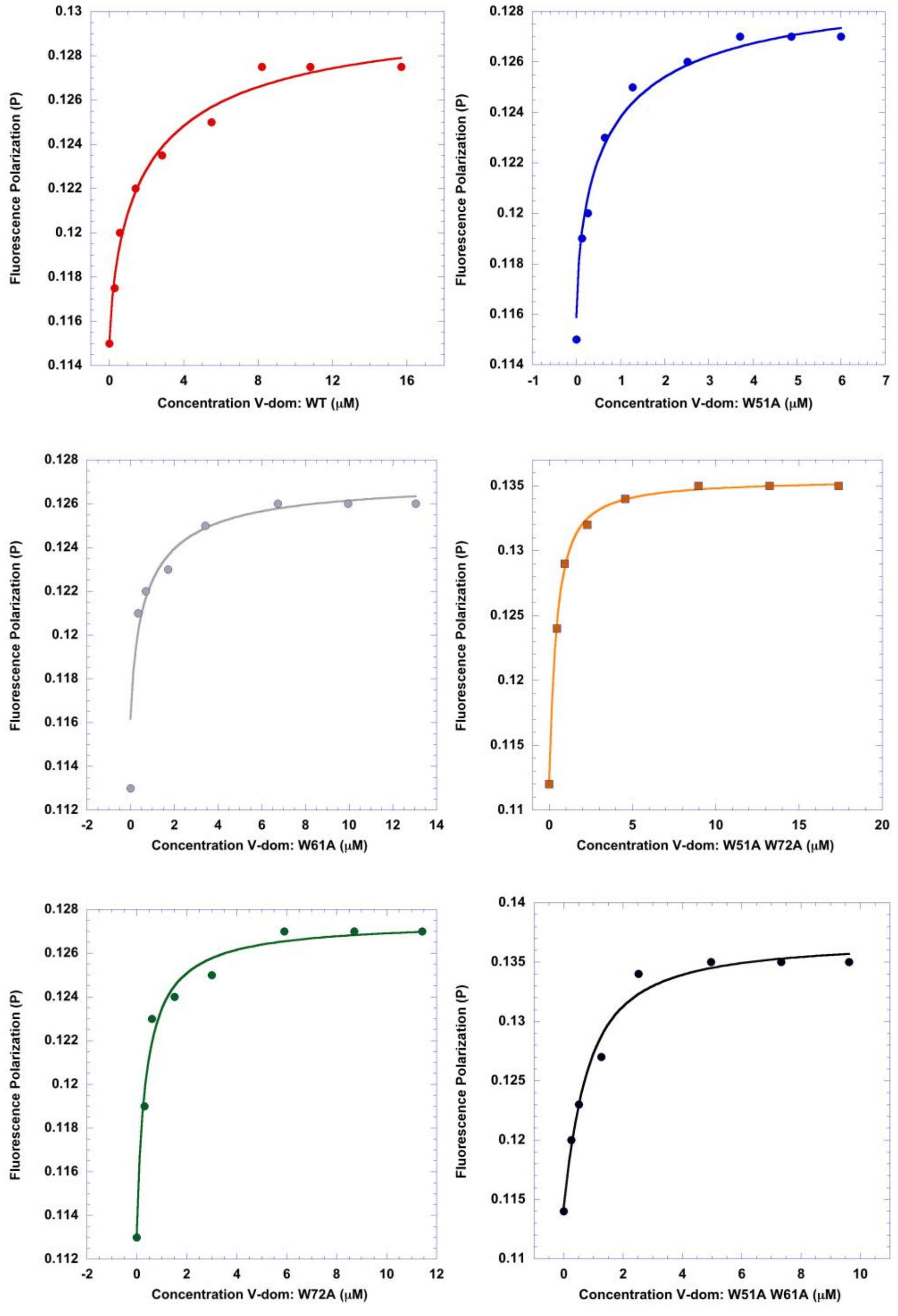

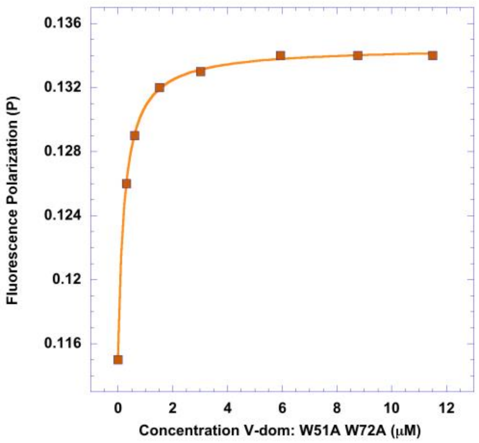

Fluorescence Polarization (P)
0.136
0.132
0.128
0.124
0.12
0.116
0
2
4
6
8
10
12
Concentration V-dom: W51A W72A (μM)

Supplemental Information: Table S1

(A) Fluorescence lifetimes folded state

Sample	τ1 (ns)	α1	τ2 (ns)	α2	τm (ns)
WT	2.33 ± 0.24	0.33 ± 0.01	6.41 ± 0.24	0.67 ± 0.01	5.08 ± 0.30
W51A	1.72 ± 0.08	0.34 ± 0.04	5.17 ± 0.10	0.66 ± 0.04	3.99 ± 0.04
W61A	1.76 ± 0.02	0.23 ± 0.02	5.91 ± 0.09	0.77 ± 0.02	4.95 ± 0.17
W72A	2.04 ± 0.03	0.28 ± 0.01	6.80 ± 0.11	0.72 ± 0.01	5.47 ± 0.09
W51A W61A	1.70 ± 0.01	0.45 ± 0.01	4.91 ± 0.10	0.55 ± 0.01	3.46 ± 0.03
W51A W72A	1.68 ± 0.03	0.37 ± 0.01	4.33 ± 0.05	0.63 ± 0.01	3.34 ± 0.05
W61A W72A	1.92 ± 0.04	0.47 ± 0.03	5.12 ± 0.17	0.53 ± 0.03	3.61 ± 0.02

(B) Fluorescence lifetimes unfolded state (6M GudHCl)

Sample	τ1 (ns)	α1	τ2 (ns)	α2	τm (ns)
WT	1.01 ± 0.02	0.36 ± 0.01	4.30 ± 0.01	0.64 ± 0.01	3.13 ± 0.02
W51A	1.00 ± 0.03	0.40 ± 0.02	4.71 ± 0.08	0.60 ± 0.02	3.24 ± 0.03
W61A	0.94 ± 0.02	0.35 ± 0.01	4.28 ± 0.13	0.65 ± 0.01	3.10 ± 0.08
W72A	1.00 ± 0.01	0.37 ± 0.01	4.40 ± 0.12	0.63 ± 0.01	3.13 ± 0.10
W51A W61A	0.87 ± 0.09	0.41 ± 0.02	4.83 ± 0.07	0.59 ± 0.02	3.19 ± 0.01
W51A W72A	0.95 ± 0.01	0.39 ± 0.02	4.70 ± 0.12	0.61 ± 0.02	3.23 ± 0.02
W61A W72A	1.03 ± 0.14	0.33 ± 0.02	4.38 ± 0.12	0.67 ± 0.02	3.28 ± 0.05

(C) Fluorescence lifetimes V-dom:S100B complex

Sample	τ1 (ns)	α1	τ2 (ns)	α2	τm (ns)
WT	1.71 ± 0.04	0.33 ± 0.04	5.51 ± 0.09	0.67 ± 0.04	4.25 ± 0.08
W51A	1.62 ± 0.10	0.35 ± 0.02	5.48 ± 0.31	0.65 ± 0.02	4.14 ± 0.15
W61A	1.74 ± 0.05	0.36 ± 0.03	5.49 ± 0.08	0.65 ± 0.03	4.15 ± 0.07
W72A	1.61 ± 0.06	0.30 ± 0.02	5.85 ± 0.09	0.70 ± 0.02	4.57 ± 0.01
W51A W61A	1.00 ± 0.12	0.34 ± 0.02	4.62 ± 0.01	0.67 ± 0.02	3.41 ± 0.10
W51A W72A	1.23 ± 0.08	0.30 ± 0.05	4.57 ± 0.17	0.70 ± 0.05	3.55 ± 0.32
W61A W72A	1.24 ± 0.17	0.20 ± 0.03	4.96 ± 0.09	0.80 ± 0.03	4.22 ± 0.16

Supplemental Table S2: Burried peptide surface areas

Protein	W72	W61	TRTK12
S100B / peptide interface surface	505 Å^2<br>S100B: 472.6<br>Peptide: 537.8	466.9 Å^2<br>S100B: 448.1<br>Peptide: 485.6	542.2 Å^2<br>S100B: 497.6<br>Peptide: 586.7
Arg 57		97.61	
Thr 58		51.92	
Glu 59		37.52	
Ala 60		79.15	
Trp 61		65.89	
Lys62		0	
Val 63		98.00	
Leu 64		36.38	
Ser 65		19.14	
		**485.61 (36.4%)**	
Pro 71	102.17		
Trp 72	164.43		
Asp 73	41.37		
Ser 74	40.11		
Val 75	72.77		
Ala 76	43.33		
Arg 77	72.54		
Val 78	0.50		
Leu 79	0.00		
	**537.22 (34.4%)**		
Thr 3			67.04
Lys 4			63.54
Ile 5			95.78
Asp 6			22.53
Trp 7			141.17
Asn 8			0
Lys 9			46.43
Ile 10			81.02
Leu 11			69.25
			**586.76 (48.1%)**
